# Catalpol Inhibits Macrophage Polarization and Prevents Postmenopausal Atherosclerosis Through Regulating Estrogen Receptor Alpha

**DOI:** 10.3389/fphar.2021.655081

**Published:** 2021-04-30

**Authors:** Qi Chen, Xu Qi, Weiwei Zhang, Yuhan Zhang, Yunhui Bi, Qinghai Meng, Huimin Bian, Yu Li

**Affiliations:** ^1^ School of Pharmacy, Nanjing University of Chinese Medicine, Nanjing, China; ^2^ Department of Respiratory Medicine, The First Affiliated Hospital of Nanjing Medical University, Nanjing, China; ^3^ Jiangsu Key Laboratory for Pharmacology and Safety Evaluation of Chinese Materia Medica, Nanjing University of Chinese Medicine, Nanjing, China; ^4^ School of Medicine and Life Sciences, Nanjing University of Chinese Medicine, Nanjing, China

**Keywords:** catalpol, postmenopausal atherosclerosis, macrophage polarization, estrogen receptor, inflammation, oxidatie stress

## Abstract

Lacking estrogen increases the risk of atherosclerosis (AS) in postmenopausal women. Inflammation plays a vital role in the pathological process of AS, and macrophages are closely related to inflammation. Catalpol is an iridoid glucoside extracted from the fresh roots of the traditional Chinese herb *Rehmanniae radix preparata*. In this study, we aimed to evaluate the effects of catalpol on macrophage polarization and postmenopausal AS. In addition, we investigated whether the mechanism of catalpol was dependent on regulating the expression of estrogen receptors (ERs). *In vitro*, lipopolysaccharides (LPS) and interferon-γ (IFN-γ) were applied to induce M1 macrophage polarization. *In vivo*, the ApoE^−/−^ mice were fed with a high-fat diet to induce AS, and ovariectomy was operated to mimic the estrogen cessation. We demonstrated catalpol inhibited M1 macrophage polarization induced by LPS and INF-γ, and eliminated lipid accumulation in postmenopausal AS mice. Catalpol not only suppressed the inflammatory response but also reduced the level of oxidative stress. Then, ERs (ERα and ERβ) inhibitors and ERα siRNA were also applied in confirming that the protective effect of catalpol was mediated by ERα, rather than ERβ. In conclusion, catalpol significantly inhibited macrophage polarization and prevented postmenopausal AS by increasing ERα expression.

## Introduction

Atherosclerosis (AS) is closely related to inflammation and the disorder of lipid metabolism (Feng et al., 2020; van Leent et al., 2021). Inflammation promotes the change of endothelial cell function Wang et al. (2013), which results in the release of pro-inflammatory factors, as well as the monocyte chemokines. The increase of pro-inflammatory factors in the vessel leads leukocytes to infiltrate into the arteries. In addition, stimulated by chemokines, the monocytes in peripheral blood migrate to the endothelium and differentiate into macrophages (Vieceli Dalla Sega et al., 2019). The activation of macrophages is relevant to the expression of inflammatory cytokines and the release of reactive oxygen species (ROS). With the decline of migration ability, the activated macrophages phagocytose modified lipoproteins and damaged cells (Orekhov et al., 2020). Then macrophages contain more cholesterol and differentiate into foam cells, which persist in plaques and promote the progress of AS (Cybulsky et al., 2016).

Macrophages play a crucial role in maintaining the homeostasis of tissue, and they can differentiate into different subtypes according to the stimulation of the microenvironment (An et al., 2021). M1 macrophages can promote inflammation by activating Toll-like receptor 4 (TLR4) related signal pathway. M1 macrophages also express a high level of pro-inflammatory cytokines such as tumor necrosis factor α (TNF-α), interleukin (IL)-1β, IL-6, ROS, and inducible nitric oxide synthase (iNOS), which aggravate the development of AS (Tabas and Bornfeldt, 2016). On the contrary, M2 macrophages release several anti-inflammatory cytokines, including IL-10 and the transforming growth factor-β (TGF-β). In the initial AS process, macrophages exhibit protective effects on lipid uptake and reduce the plaques' size. While, upon the disorder of cholesterol metabolism, more macrophages differentiate into foam cells and aggregate in the necrotic area of plaques (Moore et al., 2013).

Besides, the estrogen deficiency dramatically increases the risk of cardiovascular disease in postmenopausal women, which suggests estrogen is related to the development of AS (Meng et al., 2021). Estrogen exerts its biological effects mainly through estrogen receptors (ERs). ERα and ERβ are the classical nuclear estrogen receptors (Revankar et al., 2019). Many studies have reported that the expression of ERα can protect premenopausal women from AS (Bojar et al., 2015; Kassi et al., 2015). Estrogen also has an essential effect on the immune function of monocytes and macrophages, and it can inhibit the infiltration of macrophages during the process of AS (Calippe et al., 2010). However, hormone replacement therapy in postmenopausal women caused several adverse effects (Vinogradova et al., 2020; Szabo et al., 2021). Thus, there is an urgent need for us to find some safe estrogen-like drugs.

Catalpol, an iridoid glucoside, is extracted from the fresh roots of the traditional Chinese herb *Rehmanniae radix preparata*. Catalpol shows a wide range of biological activities, like anti-oxidation, anti-inflammation, anti-cancer, anti-apoptosis, and neuroprotection (Jiang et al., 2015; Yuan et al., 2019; Zhang et al., 2019). Firstly, Liu found that catalpol attenuated atherosclerotic lesions in a rabbit AS model by reducing inflammatory and oxidative stress (Liu and Zhang, 2015). Then, it was reported that catalpol could ameliorate diabetic AS by inhibiting neointimal hyperplasia and macrophage recruitment (Liu et al., 2016). Moreover, catalpol reduced DNA damage and prevented AS in LDLR^−/−^ mice via activating the PGC-1α/TERT pathway (Zhang et al., 2018). In human aortic endothelial cells, catalpol reduced homocysteine-induced oxidation and cell apoptosis by suppressing Nox4/NF-κB and ER stress (Hu et al., 2019). In the current study, we aimed to evaluate whether catapol alleviated postmenopausal AS through changing the phenotype of macrophages and if the mechanism was related to regulating the expression of ERs. We hope this research may provide some novel sights and evidence for catalpol in the treatment against postmenopausal AS.

## Materials and Methods

### Reagents

Catalpol (purity >98%) was purchased from the National Institute for the Control of Pharmaceutical and Biological Products (Beijing, China). Lipopolysaccharides (LPS), Interferon-γ (IFN-γ), 17β-estradiol (E_2_), methyl-piperidino-pyrazole (MPP), 4-(2-phenyl-5,7-bis [trifluoromethyl]pyrazolo [1,5-a]pyrimidin-3-yl)phenol (PHTPP) and pentobarbital sodium were obtained from Sigma-Aldrich (St. Louis, MO, United States). All Elisa kits were purchased from MultiSciences (Lianke) Biotech Co., Ltd. (Hangzhou, China), and all antibodies were obtained from Abcam (Cambridge, United Kingdom). ERα siRNA was purchased from JiKai Bio-tech Co., Ltd. (Nanjing, China).

### Animal Grouping and Drug Administration

Forty ApoE^−/−^ female mice weighing 20 ± 2 g (8 weeks old) were provided by Nanjing Biomedical Research Institute of Nanjing University. Apart from eight ApoE^−/−^ control mice (ApoE^−/−^ mice + normal diet + sham operation), thirty-two ApoE^−/−^ mice were fed with a high-fat diet (containing 0.3% cholesterol and 20% pork fat) for 90^ ^days. Thirty-two ApoE^−/−^ mice were divided into four groups, including high-fat diet group (ApoE^−/−^ mice + high-fat diet + sham operation), model group (ApoE^−/−^ mice + high-fat diet + ovariectomy), 17β-estradiol (E_2_) (0.13 mg/kg via i. g.) group (ApoE^−/−^ mice + high-fat diet + ovariectomy + E_2_), and Catalpol (20 mg/kg via i. g.) group (ApoE^−/−^ mice + high-fat diet + ovariectomy + Catalpol). The doses of administration were according to our previous research (Jing et al., 2017). To establish the postmenopausal AS model, we carried sterile ovary ligation and ovariectomy (Ovx) on mice a week before oral administration. While mice in ApoE^−/-^ control group and high-fat diet group received sham operation. All mice were anesthetized by injecting intraperitoneally with pentobarbital sodium. The levels of TC, TG, HDL-c, LDL-c, and NO in the serum were determined by kits (Nanjing Jiancheng Bioengineering Institute, Nanjing, China). The levels of iNOS, eNOS, CRP, IL-1 β, TNF-α, IL-10, and CD11b in the serum were determined by ELISA kits (Nanjing Boshuo Biotechnology Co., Ltd., Nanjing, China).

The animal experiments were performed according to the Guidelines and Policies for Animal Surgery provided by Nanjing University of Chinese Medicine. The study was approved by the Institutional Ethics Committee of Animal Care (A171002).

### Quantification of Atherosclerotic Lesions

Hematoxylin-eosin (H&E) and oil red O were applied to stain the 8 μm-thick sections of the aortic root. The atherosclerotic lesion was quantified as a relative percentage using Image-Pro Plus 6.0 software (Qin et al., 2015).

### Isolation of Murine Peritoneal Macrophage

The peritoneal macrophage of all mice was isolated according to the published method (Zhang et al., 2008), and it was identified through Wright’s staining. Briefly, 1 ml 3% Brewer thioglycollate medium were injected into the mice peritoneal cavity 24 h before sacrifice. The next day, all mice were soaked with 70% alcohol. Then, a 10 ml cold harvest medium was inserted through the mouse’s peritoneal wall. After 5 min, the peritoneal fluid was withdrawn slowly and dispensed into a 15 ml centrifuge tube on ice. The peritoneal fluid was centrifuged at 1,000 rpm for 10 min, and the supernatant was discarded. The bottom cells were washed with PBS buffer and resuspended by 8 ml DMEM medium with 20% fetal bovine serum. After incubating for 6 h, the adherent cells were peritoneal macrophages.

### Immunohistochemistry

The aorta sections were incubated with rabbit anti-mouse ERα and ERβ polyclonal antibody (1:100) for 2 h at 37°C. After rinsing, the sections were incubated with peroxidase-conjugated affinipure goat anti-mouse IgG antibody (1:100) for 30 min at room temperature and then with 0.5 g/L diaminobenzidine for 5 min. Images were taken at five random fields (× 100) and quantified with the Image-Pro Plus 6.0 software.

### Cell Culture and Grouping

The mouse macrophage cell line J774A-1 was purchased from the Cell Bank of the Chinese Academy of Science (Shanghai, China). J774A-1 cells were cultured in Dulbecco’s modified Eagle’s medium (DMED) supplemented with 10% fetal bovine serum (FBS), 100 kU/L benzylpenicillin, and 100 mg/L streptomycin. The mixture of LPS (100 ng/ml) and IFN-γ (20 ng/ml) was used to treat cells (Cutone et al., 2017). E_2_ group was treated with 100 nM E_2_ (Meng et al., 2020), and catalpol groups were treated with three different concentrations at 5, 10, and 20 μM catalpol. Besides, both ERα inhibitor MPP, ERα siRNA, and ERβ inhibitor PHTPP were used to establish the ERα or ERβ low-expression cell model.

### Cell Viability Assay

J774A-1 cells (1 × 10^4^/well) were seeded in a 96-well microplate and serum-starved for 16 h when they reached 60% confluence. After the treatment, MTT was dissolved in PBS at a concentration of 5 mg/ml 200 μL MTT solution was added to each well, and the plate was subsequently incubated for 4 h. In the end, 150 μL DMSO was added to each well to dissolve formazan crystals, and the absorbance was measured at 570 nm.

### Western Blot Analysis

According to the instruction, the concentration of protein was detected by the BCA assay kit (Beyotime, Shanghai, China). Then 30 μg proteins were separated by 10% SDS-PAGE and were transferred to PVDF membranes. After blocking, the primary antibodies were incubated with membranes at 4°C overnight. Then the secondary antibody was used to incubate membranes for 90 min. In the end, the target proteins were determined with an ECL system (Millipore, Billerica, MA, United States) and visualized by a ChemiDoc XRS system (Bio-Rad, Hercules, CA, United States). The relative expression levels of the target proteins were determined with Image Lab software.

### Real-Time Polymerase Chain Reaction

The mRNA expression M1 markers (iNOS, CD86) and M2 markers (Arg1, CD206) were detected by the quantitative RT-PCR. TRIzol reagent (Invitrogen, Carlsbad, CA, United States) was used to isolate the total RNA of J774A-1 cells. RNA was reversely transcribed into cDNA by the First Strand cDNA Synthesis kit (Thermo Fisher Scientific, Waltham, MA, United States). The primer sequences were presented in Supplymentary Table S1. The Real-time PCR reaction was amplified using ABI QuantStudio6 Q6 with AceQ qPCR SYBR^®^ Green Master Mix (Vazyme, Nanjing, China).

### Immunofluorescence

Followed by permeabilization with 0.5% Triton X-100, J774A-1 cells were fixed with 4% paraformaldehyde. Cells were blocked with 1% BSA and incubated with different antibodies at 4°C overnight. Then, the fluorescence-labeled secondary antibody was applied to treat cells, and the expression of fluorescence was photographed by a Zeiss inverted microscope with the ZEN 2011 imaging software.

### Fluorescence-Activated Cell Sorting

J774A-1 cells (1 × 10^4^/well) were seeded in a 6-well microplate and serum-starved for 16 h when they reached 60% confluence. After the treatment, the cells were collected and washed by PBS three times. Then, the cells were fixed and permeabilized by a Fixation and Permeabilization Kit (4 A Biotech, Beijing, China). After the permeabilization, the cells were centrifuged at 1,000 rpm for 5 min, and 100 μL PBS was used to resuspend cells. Subsequently, PE-CD86 and APC-CD163 (BioLegend, San Diego, CA, United States) antibodies were added to the cell suspension. After 40 min of incubation in the dark, the cells were washed with PBS and subjected to FACS. FACS was performed and analyzed by Beckman Gallios (Beckman Coulter, Fullerton, CA, United States).

### Statistical Analysis

The data were statistically analyzed by one-way ANOVA followed by Tukey’s multiple comparison tests employing Prism (Version 6.0; GraphPad Software Inc., San Diego, CA, United States). Data are expressed as mean ± SD. Statistical significance was accepted at the *p* value of <0.05.

## Results

### LipopolysaccharidesCatalpol Inhibited M1 Macrophage Polarization Induced by and Interferon-γ

**FIGURE 1:**
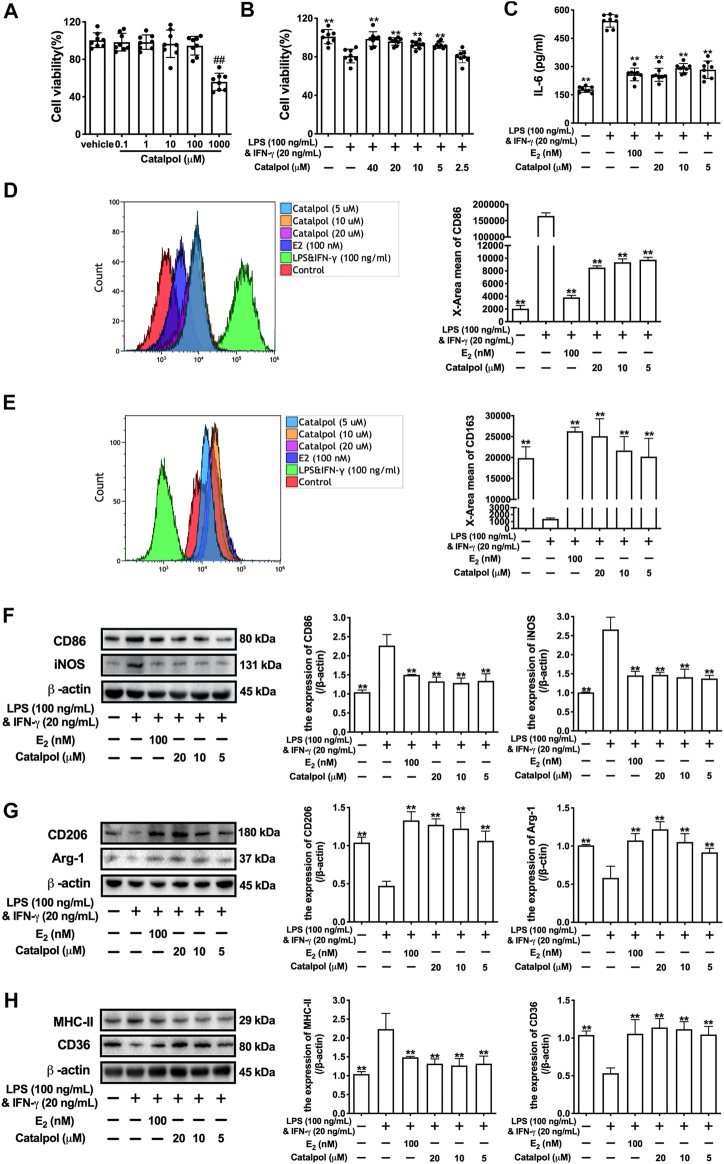
Catalpol inhibited M1 macrophage polarization induced by LPS and INF-γ. **(A)** J774A-1 were treated with different concentrations of catalpol (*n* = 8); **(B)** Effects of catalpol on M1 macrophage induced by LPS (100 ng/ml) and INF-γ (20 ng/ml) (*n* = 8); **(C)** Effects of catalpol on the level of IL-6 (*n* = 8); **(D)** The level of M1-macrophage marker CD86 was analyzed by FACS (*n* = 3); **(E)** The level of M2-macrophage marker CD163 was analyzed by FACS (*n* = 3); **(F)** Effects of catalpol on the expressions of M1 macrophage markers (CD86 and iNOS) (*n* = 3); **(G)** Effects of catalpol on the expressions of M2 macrophage markers (CD206 and Arg-1) (*n* = 3); **(H)** Effects of catalpol on the expressions of MHC-II and CD36 (*n* = 3). Data are expressed as mean ± SD. #*p* < 0.05, ##*p* < 0.01 vs. vehicle group; *p < 0.05, **p < 0.01 vs. LPS&INF-γ group.

Firstly, the cytotoxicity of catalpol on J774A-1 was detected. As shown in Figure 1A, 1,000 μM catalpol dramatically inhibited the growth of J774A-1, and catalpol with the concentrations under 100 μM was applied in the following experiments. Then, 100 ng/ml LPS and 20 ng/ml INF-γ were combined to induce the M1 macrophage polarization. Compared to the vehicle group, the cell viability of J774A-1 was declined by 20% after treated with LPS&INF-γ for 24 h. While catalpol (40, 20, 10, and 5 μM) could promote the growth of cells treated with LPS&INF-γ (Figure 1B). In addition, the pro-inflammatory cytokine IL-6 secreted by J774A-1 increased significantly after the treatment with LPS&INF-γ, and the level of IL-6 was effectively attenuated by E_2_ and catalpol (Figure 1C). It implied that catalpol suppressed inflammation in LPS&INF-γ treated macrophages.

To determine the effect of catalpol on macrophage polarization, we detect the level of M1 macrophage marker CD86 (Figure 1D) and M2 macrophage marker CD163 (Figure 1E) by FACS. The result showed that LPS&INF-γ significantly increased CD86 and decreased CD163, which implied the M1 macrophage polarization was caused by LPS&INF-γ. While the polarization was reversed by E_2_ and catalpol. We also carried western blot to confirm the expressions of macrophage markers. As shown in Figure 1F, the expressions of M1 macrophage markers (CD86 and iNOS) dramatically up-regulated in LPS&INF-γ treated cells. Both E_2_ and catalpol reduced the expressions of CD86 and iNOS, but the protective effects of catalpol were not in a dose-dependent pattern. Catalpol also reversed the mRNA levels of CD86 and iNOS induced by LPS&INF-γ (Supplementary Figures S1A,B). On the contrary, LPS&INF-γ caused a dramatic decline in the expressions of M2 macrophage markers (CD206 and Arg-1). In contrast, CD206, and Arg-1 were up-regulated by the treatment with E_2_ and catalpol (Figure 1G). Catalpol also promoted the mRNA level of CD206 and Arg-1 in M1 macrophages (Supplementary Figures S1C,D).

To evaluate the effect of catalpol on macrophages’ functions, we detected the expressions of MHC-II and CD36. MHC-II is a binding partner of CD4 + T cells, which can stimulate the production of INF-γ. As shown in Figure 1H, LPS&INF-γ significantly increased the expression of MHC-II, and it was reversed by E_2_ and catalpol. In addition, catalpol increased the expression of lipid transport-associated protein CD36 in LPS&INF-γ treated cells (Figure 1H). The results implied that catalpol could improve the function of classically activated macrophages.

### Catalpol Attenuated Inflammatory Response and Oxidative Stress in M1 Macrophages

**FIGURE 2:**
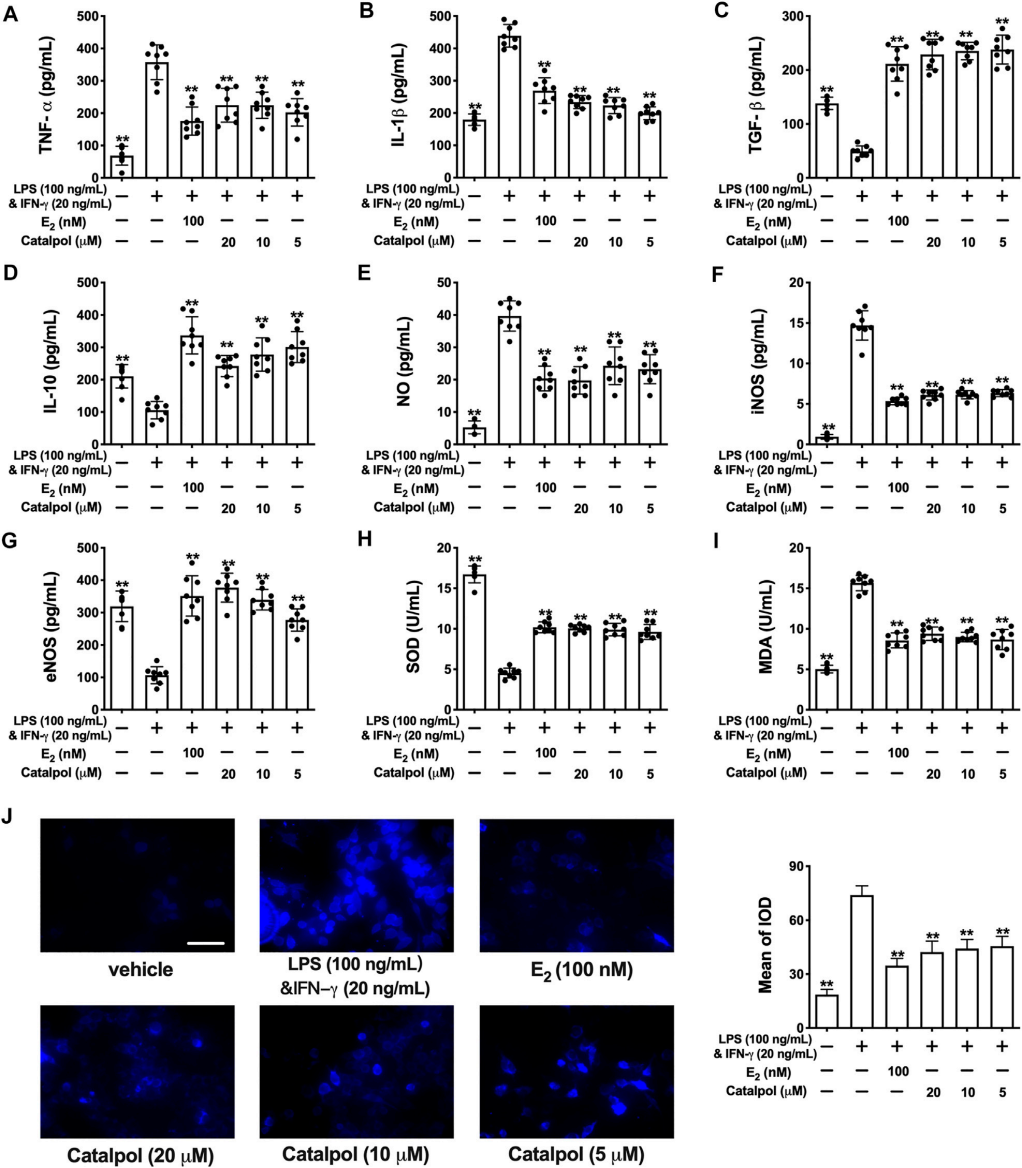
Catalpol attenuated inflammatory response and oxidative stress in M1 macrophages. **(A, B)** Effects of catalpol on the levels of pro-inflammatory cytokines (TNF-α and IL-1β) (*n* = 8); **(C, D)** Effects of catalpol on the levels of anti-inflammatory cytokines (TGF-β and IL-10) (*n* = 8); **(E, G)** Effects of catalpol on the levels of NO, iNOS and eNOS (*n* = 8); **(H, I)** Effects of catalpol on the levels of SOD and MDA (*n* = 8); **(J)** Effect of catalpol on the level of ROS was stained by immunofluorescence (*n* = 3). Data are expressed as mean ± SD. *p < 0.05, **p < 0.01 vs. LPS&INF-γ group.

As shown in Figures 2A,B fig2, the released pro-inflammatory cytokines (TNF-α and IL-1β) increased dramatically in M1 macrophages induced by LPS&INF-γ. Both E_2_ and catalpol could reduce the content of TNF-α and IL-1β, but the levels were still higher than in the vehicle group. Instead, LPS&INF-γ significantly down-regulated the release of anti-inflammatory cytokines, including TGF-β and IL-10 (Figures 2C,D). Notably, TGF-β and IL-10 in E_2_ and catalpol treated cells were much higher than in the vehicle group. It suggested that catalpol could attenuate the inflammatory response in M1 macrophages, as well as E_2._

Oxidative stress plays a vital role in macrophage activation and foam cell formation. Compared to the vehicle group, the level of NO was promoted in M1 macrophages. LPS&INF-γ also up-regulated the release of iNOS and down-regulated the release of eNOS. While E_2_ and catalpol significantly increase the level of eNOS and reduce the level of iNOS and NO in M1 macrophages (Figures 2E–G). Also, LPS&INF-γ caused the decrease of SOD and the increase of MDA in M1 macrophages, which were reversed by E_2_ and catalpol (Figures 2H,I). As shown in Figure 2J, E_2_ and catalpol significantly inhibited the level of ROS induced by LPS&INF-γ. In summary, catalpol exhibited the effect of reducing oxidative stress in M1 macrophages.

### Catalpol Inhibited M1 Macrophage Polarization Through Regulating Estrogen Receptors α

**FIGURE 3:**
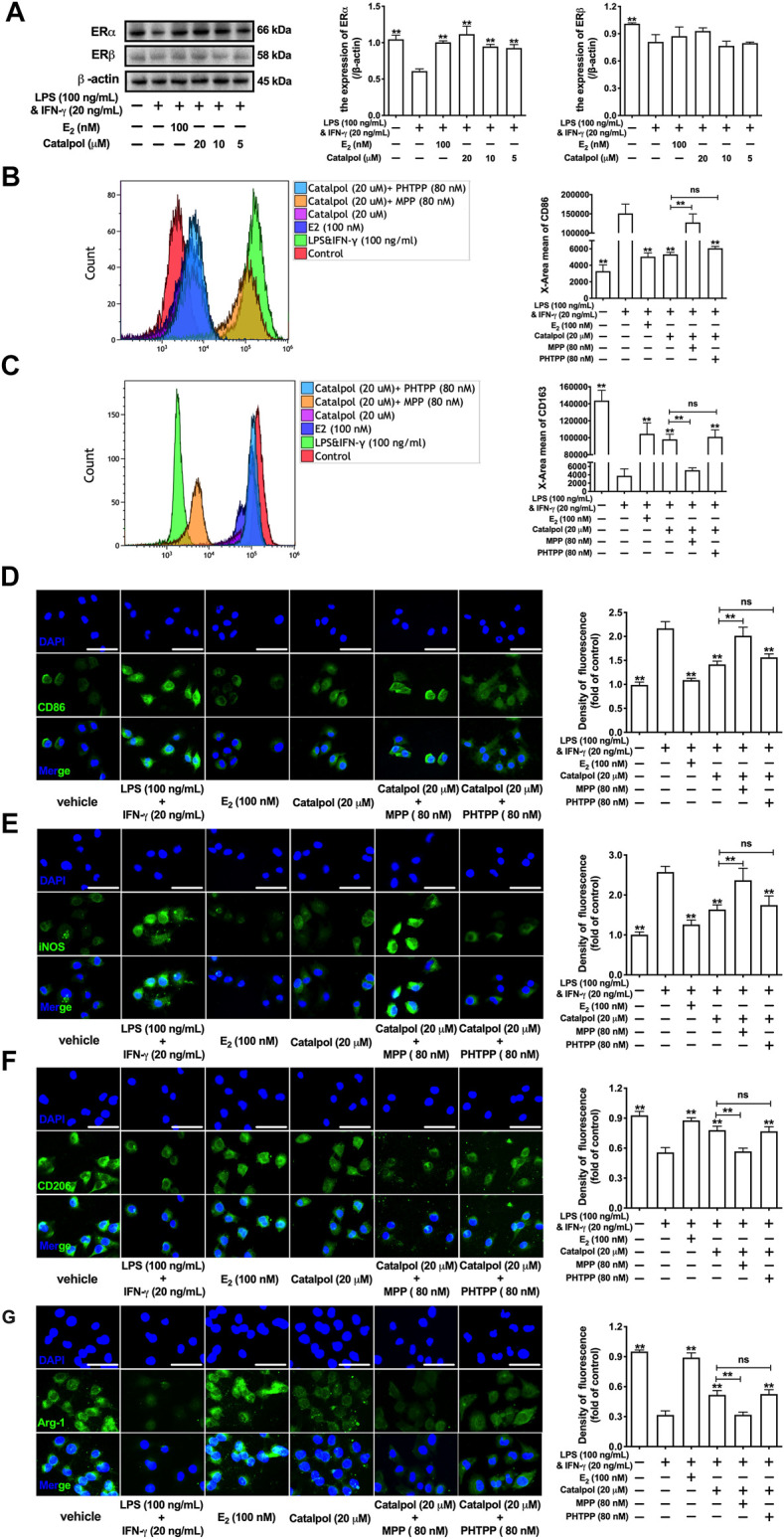
Catalpol inhibited M1 macrophage polarization through regulating ERα. **(A)** Effects of catalpol on the expressions of ERα and ERβ (*n* = 3); **(B, C)** With MPP and PHTPP, the level of M1 macrophage markers CD86 and M2 macrophage markers CD163 were detected by FACS (*n* = 3); **(D, E)** With MPP and PHTPP, effects of catalpol on the expressions of M1 macrophage markers (CD86 and iNOS) were stained by immunofluorescence (*n* = 3); **(F, G)** With MPP and PHTPP, effects of catalpol on the expressions of M2 macrophage markers (CD206 and Arg-1) were stained by immunofluorescence (*n* = 3).

The expressions of ERs were assessed by western blot. Compared to the vehicle group, LPS&INF-γ significantly reduce the expression of ERα, as well as ERβ. However, the treatment with catalpol could only increase the ERα expression in M1 macrophages, rather than ERβ (Figure 3A). To confirm the effect of catalpol was mediated by ERs, both ERα inhibitor (MPP) and ERβ inhibitor (PHTPP) were involved in our experiments. The result of FACS demonstrated that catalpol inhibited M1 macrophage marker CD86, and the decline was reversed by the co-treatment with MPP, rather than PHTPP (Figure 3B). Meanwhile, MPP blocked the promotion of M2 macrophage marker CD163 caused by catalpol. With the co-treatment of PHTPP, catalpol could also increase the level of CD163 in LPS&INF-γ-treated macrophages (Figure 3C). It was implied that catalpol suppressed M1 macrophage by ERα, rather than ERβ. Then, we also carried immunofluorescence to verify this hypothesis. As shown in Figures 3D,E, catalpol could reduce the expression of CD86 and iNOS in M1 macrophages. The combination of MPP reversed the declines caused by catalpol, while the expressions of CD86 and iNOS were still significantly lowered by the co-treatment with PHTPP. Similarly, MPP blocked the increase of M2 macrophage markers (CD206 and Arg-1) caused by the treatment with catalpol. Compared to the catalpol group, PHTPP did not lead to any dramatic changes in the expressions of CD206 and Arg-1 (Figures 3F,G). The mRNA levels of CD86, iNOS, CD206, and Arg-1 exhibited the same results (Supplementary Figures S2A–D).

**FIGURE 4:**
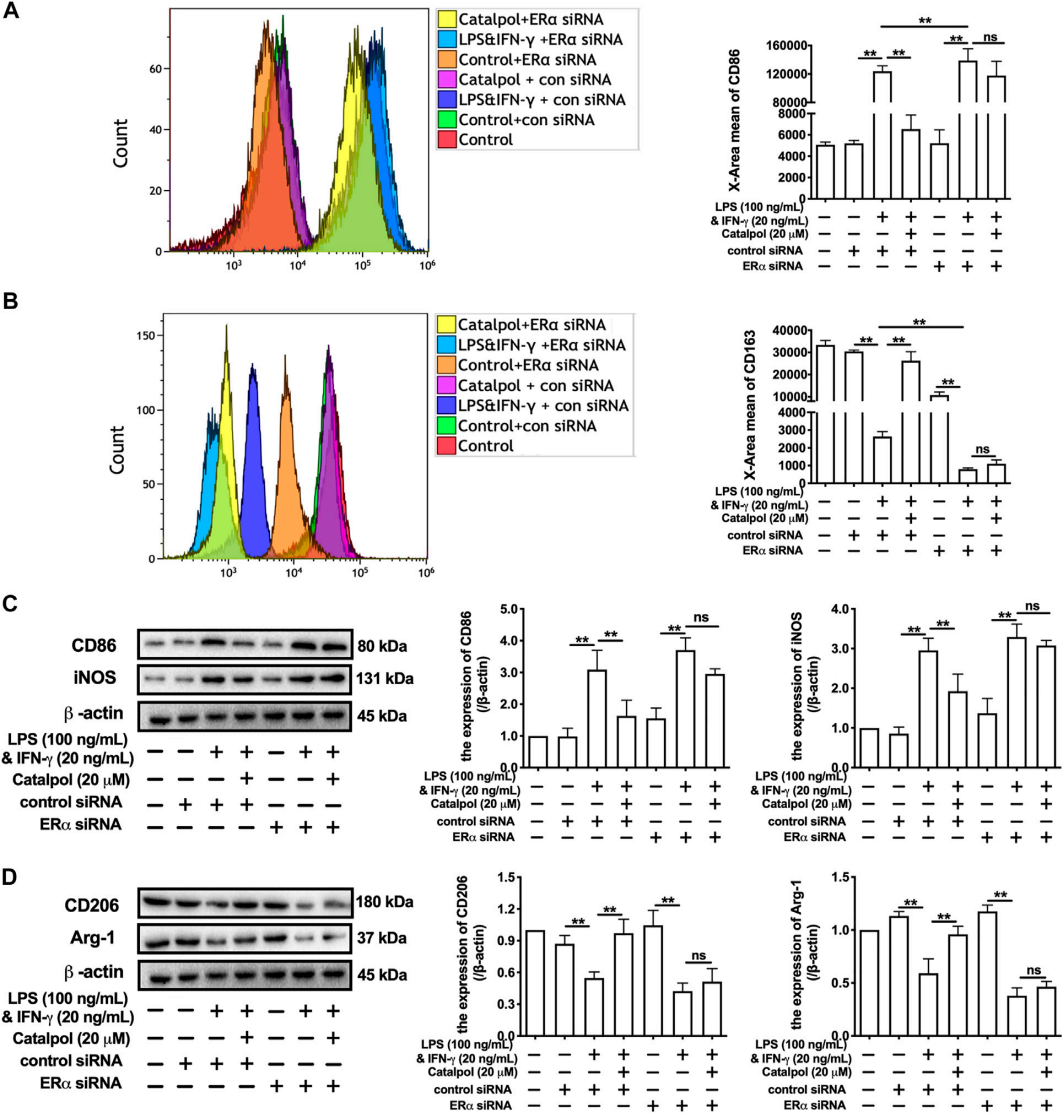
Catalpol inhibited M1 macrophage polarization through regulating ERα. **(A, B)** With ERα siRNA, the level of M1 macrophage markers CD86 and M2 macrophage markers CD163 were detected by FACS (*n* = 3); **(C)** With ERα siRNA, effects of catalpol on the expressions of M1 macrophage markers (CD86 and iNOS) were detected by western blot (*n* = 3); **(D)** With ERα siRNA, effects of catalpol on the expressions of M2 macrophage markers (CD206 and Arg-1) were detected by western blot (*n* = 3). Data are expressed as mean ± SD. *p < 0.05, **p < 0.01 vs. LPS&INF-γ group.

In addition, ERα siRNA was used to suppress the expression of ERα. With the lack of ERα, the effect of catalpol on reducing CD86 (Figure 4A) and increasing CD163 (Figure 4B) disappeared. The results of western blot showed that catalpol could not reduce M1 macrophage markers (CD86 and iNOS) and increase M2 macrophage markers (CD206 and Arg-1) in ERα siRNA transfected cells (Figures 4C,D). After the transfection with ERα siRNA, the mRNA levels of CD86 and iNOS could not be reduced by catalpol, and the mRNA levels of CD206 and Arg-1 could not be increased as well (Supplementary Figures S2E–H). In summary, these results implied catalpol inhibited M1 macrophage polarization through increasing ERα expression.

### Catalpol Attenuated Inflammatory Response in M1 Macrophages Through Regulating Estrogen Receptors α

**FIGURE 5:**
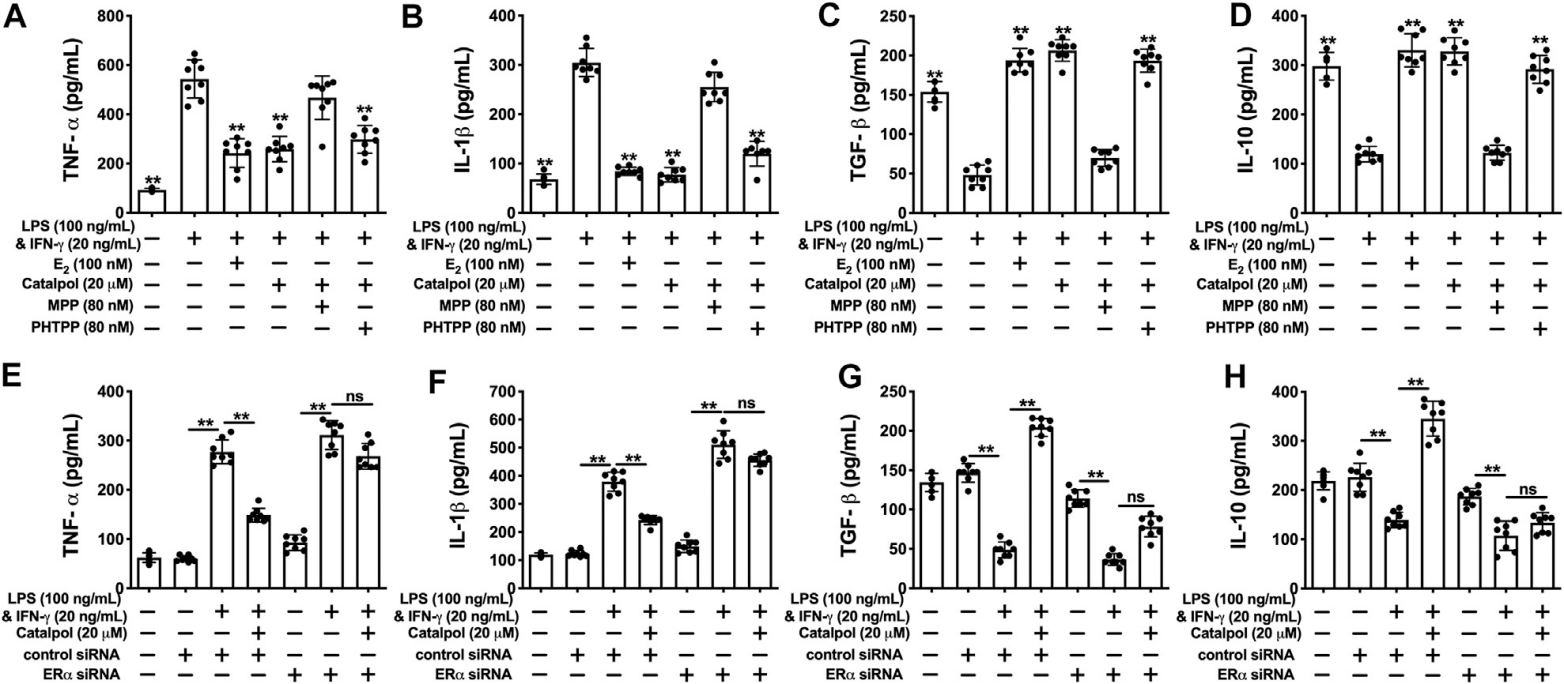
Catalpol attenuated inflammatory response in M1 macrophages through regulating ERα. **(A, B)** With MPP and PHTPP, effects of catalpol on the levels of pro-inflammatory cytokines (TNF-α and IL-1β) (*n* = 8); **(C, D)** With MPP and PHTPP, effects of catalpol on the levels of anti-inflammatory cytokines (TGF-β and IL-10) (*n* = 8); **(E, F)** ERα siRNA, effects of catalpol on the levels of pro-inflammatory cytokines (TNF-α and IL-1β) (*n* = 8); **(G, H)** With ERα siRNA, effects of catalpol on the levels of anti-inflammatory cytokines (TGF-β and IL-10) (*n* = 8). Data are expressed as mean ± SD. *p < 0.05, **p < 0.01 vs. LPS&INF-γ group.

As shown in Figures 5A,B, catalpol dramatically suppressed the levels of pro-inflammatory cytokines (TNF-α and IL-1β). MPP reversed the decreases of pro-inflammatory cytokines induced by catalpol in M1 macrophages, and there was no significant difference compared to LPS&INF-γ group. Besides, the levels of TNF-α and IL-1β were still significantly decreased by the co-treatment with catalpol and PHTPP. Similarly, the increases of anti-inflammatory cytokines (TGF- β and IL-10) induced by catalpol were blocked by MPP, rather than PHTPP (Figures 5C,D). In ERα siRNA transfected cells, catalpol could not down-regulate pro-inflammatory cytokines (Figures 5E,F) and up-regulate anti-inflammatory cytokines (Figures 5G,H).

### Catalpol Attenuated Oxidative Stress in M1 Macrophages Through Regulating Estrogen Receptors α

**FIGURE 6:**
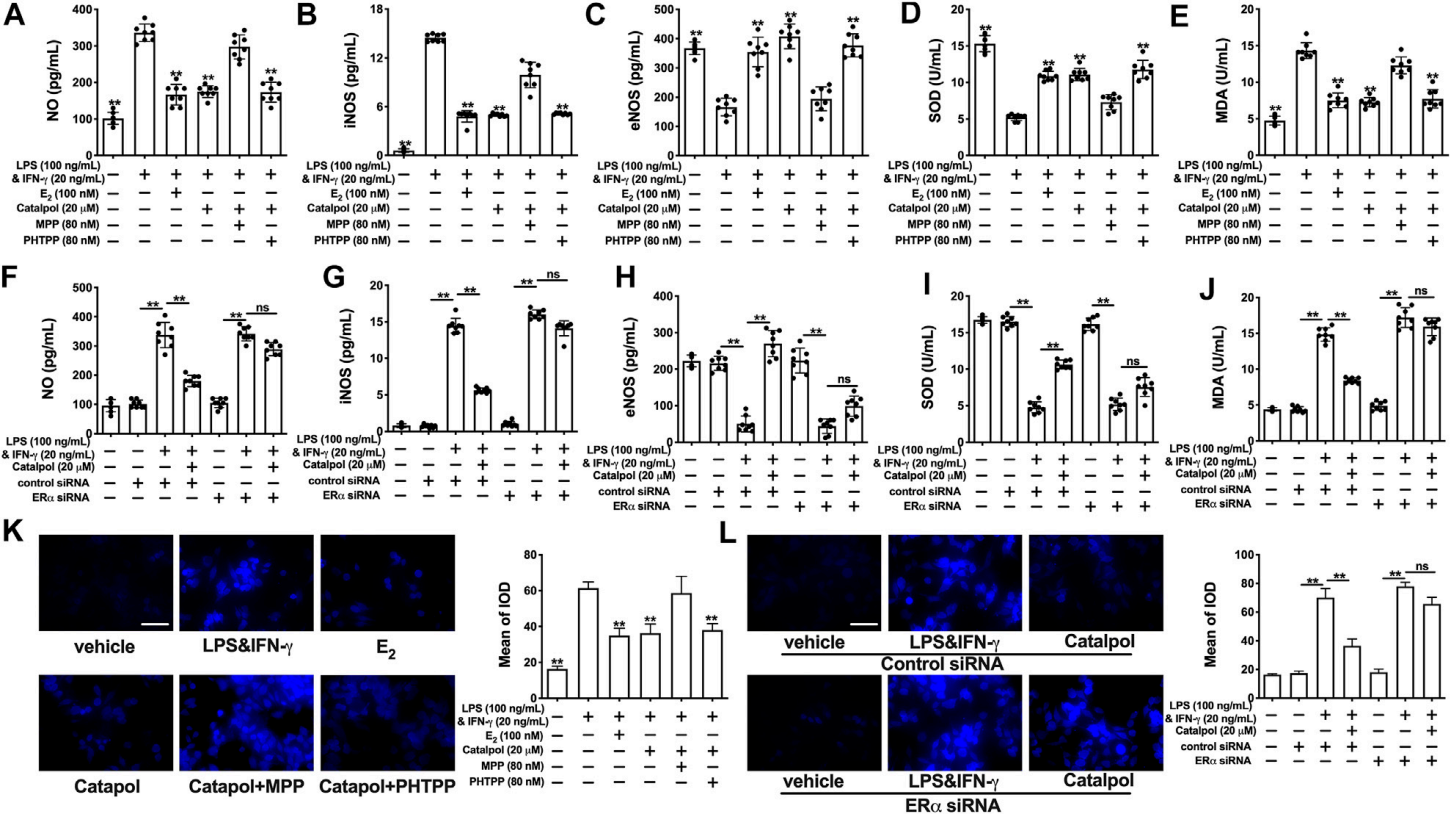
Catalpol attenuated oxidative stress in M1 macrophages through regulating ERα. **(A–C)** With MPP and PHTPP, effects of catalpol on the levels of NO, iNOS and eNOS (*n* = 8); **(D–E)** With MPP and PHTPP, effects of catalpol on the levels of SOD and MDA (*n* = 8); **(F–H)** With ERα siRNA, effects of catalpol on the levels of NO, iNOS and eNOS (*n* = 8); **(I–J)** With ERα siRNA, effects of catalpol on the levels of SOD and MDA (*n* = 8); **(K)** With MPP and PHTPP, effect of catalpol on the level of ROS was stained by immunofluorescence (*n* = 3); **(L)** With ERα siRNA, effect of catalpol on the level of ROS was stained by immunofluorescence (*n* = 3). Data are expressed as mean ± SD. *p < 0.05, **p < 0.01 vs. LPS&INF-γ group.

Catalpol inhibited the total concentration of NO in M1 macrophages, while the effect was blocked by MPP (Figure 6A) and ERα siRNA (Figure 6F). Compared to the catalpol group, MPP increased the level of iNOS, as well as decreasing the level of eNOS. There were no significant differences between the MPP group and LPS&INF-γ group (Figures 6B,C). Similarly, catalpol could not reduce iNOS and promote eNOS in ERα siRNA transfected cells (Figures 6G,H). As shown in Figures 6D–I, the concentration of SOD was up-regulated by catalpol, and the effect was blocked by MPP and ERα siRNA. The combination of MPP and catalpol could not reduce the level of MDA in M1 macrophages (Figure 6E). The effect of catalpol on reducing MDA was also blocked by ERα siRNA (Figure 6J). In addition, the results of immunofluorescence showed that the increase of ROS induced by LPS&INF-γ could not be reversed by catalpol when cells were co-treated with MPP (Figure 6K) and ERα siRNA (Figure 6L). In conclusion, the increase of ERα expression was required for catalpol to inhibit oxidative stress in M1 macrophages.

### Catalpol Reduced Lipid Accumulation in HFD-Ovariectomy-Treated ApoE^−/−^ Mice

**FIGURE 7:**
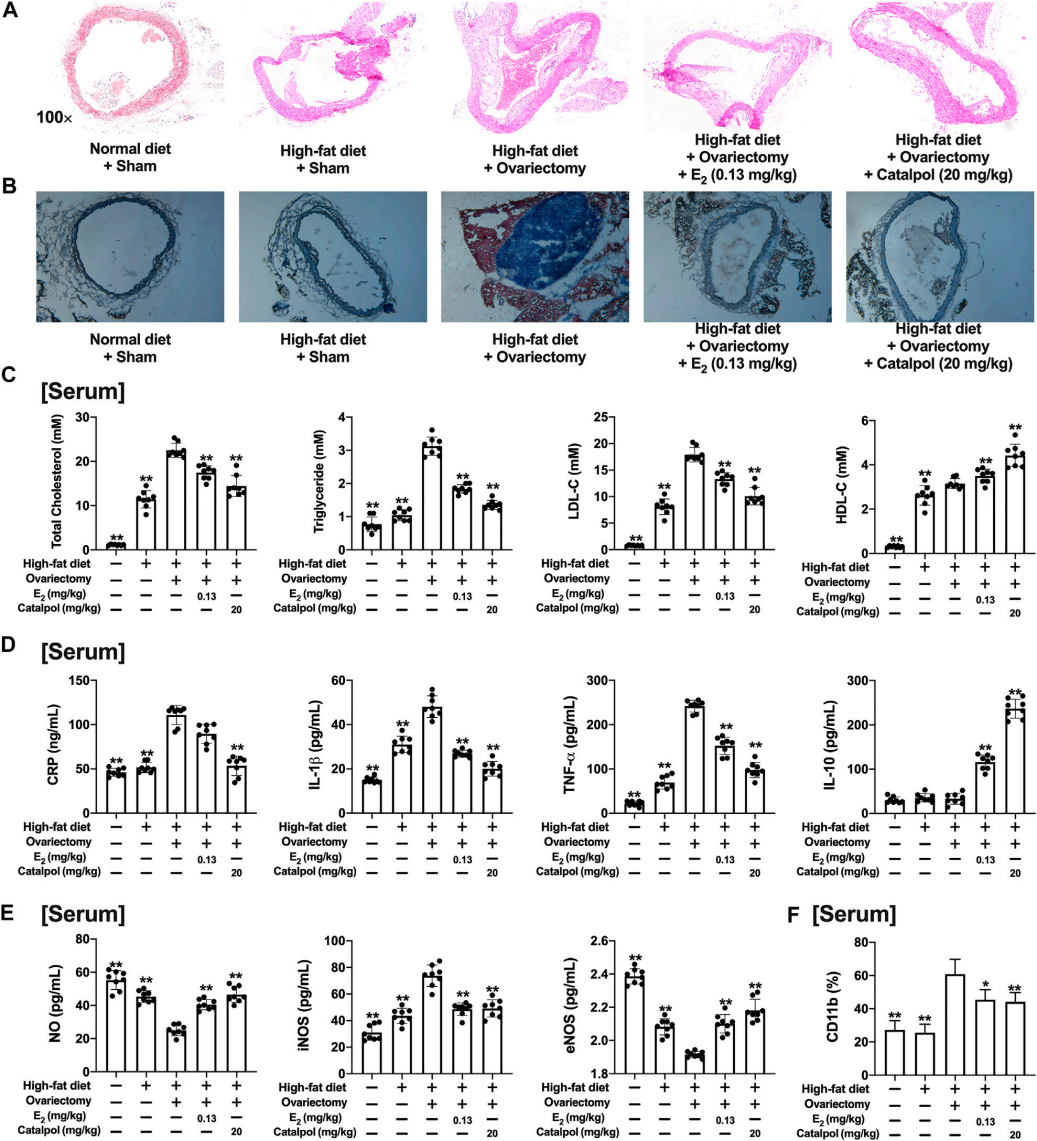
Catalpol attenuated inflammatory response and oxidative stress in HFD-Ovx-treated ApoE^−/−^ mice. Representative atherosclerotic lesions in cross-sections of the aortas were stained by H&E **(A)** and oil red O **(B)**; **(C)** Effects of catalpol on the levels of serum lipid (TC, TG, LDL-C, and HDL-C) after 90 days (*n* = 8); **(D)** Effects of catalpol on the levels of inflammatory cytokines (CRP, IL-1β, TNF-α, and IL-10) in HFD-Ovx-treated ApoE^−/−^ mice (*n* = 8); **(E)** Effects of catalpol on the levels of NO, iNOS, and eNOS in HFD-Ovx-treated ApoE^−/−^ mice (*n* = 8); **(F)** Effect of catalpol on the level of CD11b in HFD-Ovx-treated ApoE^−/−^ mice (*n* = 8); Data are expressed as mean ± SD. *p < 0.05, **p < 0.01 vs. Ovx group.

As shown in Figures 7A,B, the sections of aortas were stained to evaluate the atherosclerotic lesions. Massive stained aortic lesions appeared with a high-fat diet, and the plaque areas were increased further in the Ovx group. However, both E_2_ and catalpol could apparently reduce atherosclerotic lesions. The serum lipids of the mice were recorded (Figure 7C). The high-fat diet increased the levels of TG, TC, and LDL-C, and the levels were dramatically up-regulated after ovariectomy. However, the levels of TG, TC, and LDL-C were significantly lowered by E_2_ and catalpol. Due to the physiological stress, the HDL-C level was up-regulated after ovariectomy, and it was increased further by E_2_ and catalpol.

### Catalpol Inhibited Inflammatory Response and Oxidative Stress in HFD-Ovariectomy-Treated ApoE^−/−^ Mice

The concentrations of pro-inflammatory cytokines (CRP, TNF-α, and IL-1β) and anti-inflammatory cytokine (IL-10) in the serum were detected. As shown in Figure 7D, the high-fat diet up-regulated TNF-α and IL-1β, which was increased further in the Ovx group. E_2_ and catalpol significantly reduced TNF-α and IL-1β in HFD-Ovx-treated ApoE^−/−^ mice. The level of CRP was only promoted by ovariectomy, and it was effectively attenuated by catalpol. Notably, both a high-fat diet and ovariectomy did not cause any change in the level of IL-10. While E_2_ and catalpol dramatically increase IL-10. The level of IL-10 in the catalpol group was much higher than in the E_2_ group. The results implied catalpol inhibited the inflammatory response in HFD-Ovx-treated ApoE^−/−^ mice.

In addition, the level of NO in the serum was reduced by the high-fat diet, and it was decreased further in HFD-Ovx-treated ApoE^−/−^ mice. But, the decline of NO was reversed by E_2_ and catalpol. A high-fat diet and ovariectomy up-regulated the level of iNOS, and it was significantly reduced by E_2_ and catalpol. Conversely, E_2_ and catalpol effectively promoted the level of eNOS in HFD-Ovx-treated ApoE^−/−^ mice. In conclusion, catalpol inhibited the oxidative stress in HFD-Ovx-treated ApoE^−/−^ mice (Figure 7E).

### Catalpol Attenuated M1 Macrophage Markers and Increased M2 Macrophage Markers in HFD-Ovariectomy-Treated ApoE^−/−^ Mice

**FIGURE 8:**
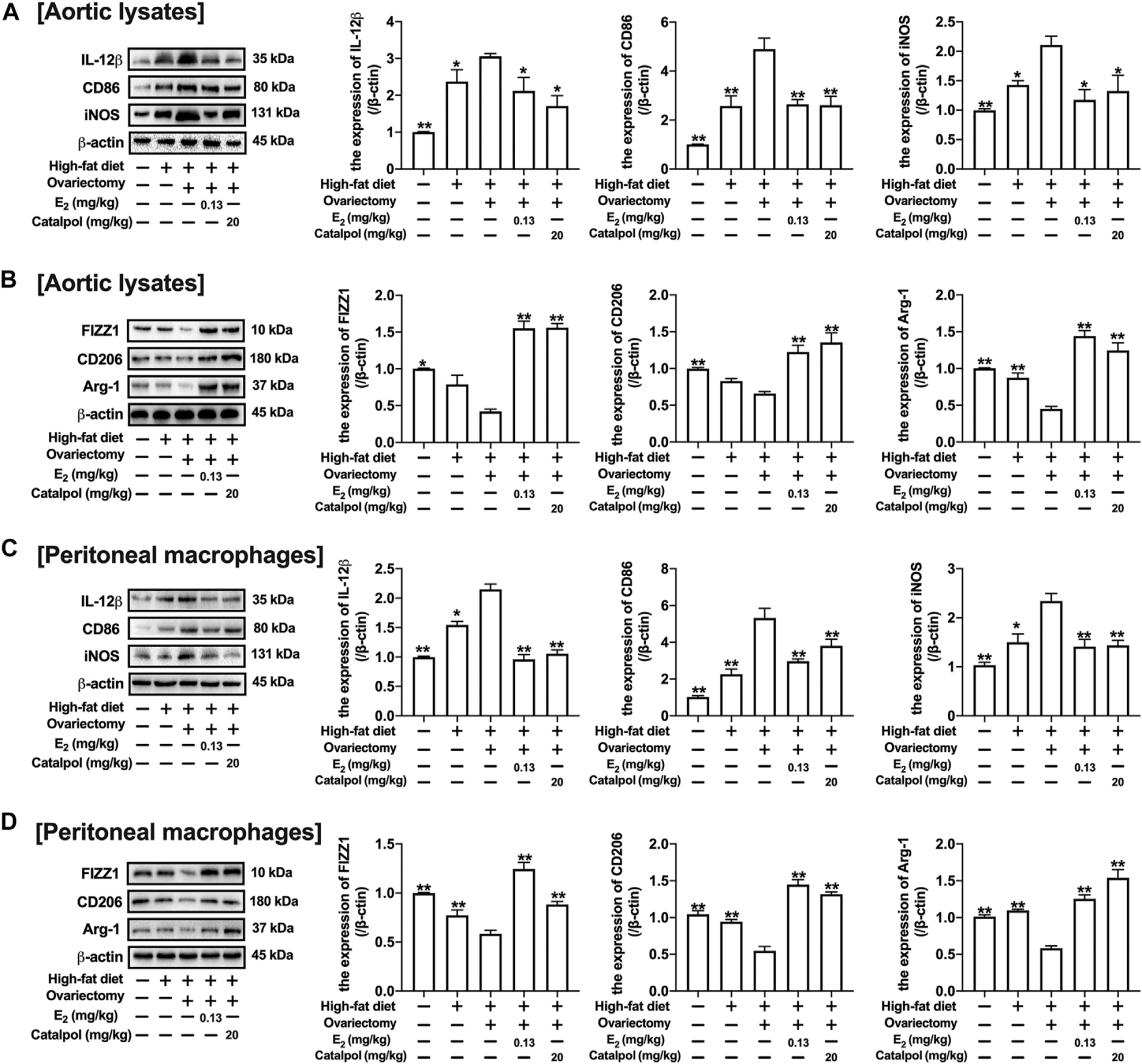
Catalpol attenuated the expression of M1 macrophage markers in HFD-Ovx-treated ApoE^−/−^ mice. **(A)** Effects of catalpol on the expressions of M1 macrophage markers (IL-12β, CD86 and iNOS) in aortic macrophages (*n* = 3); **(B)** Effects of catalpol on the expressions of M2 macrophage markers (FIZZ, CD206 and Arg-1) in aortic macrophages (*n* = 3); **(C)** Effects of catalpol on the expressions of M1 macrophage markers (IL-12β, CD86, and iNOS) in peritoneal macrophages (*n* = 3); **(D)** Effects of catalpol on the expressions of M2 macrophage markers (FIZZ1, CD206, and Arg-1) in peritoneal macrophages (*n* = 3). Data are expressed as mean ± SD. *p < 0.05, **p < 0.01 vs. Ovx group.

CD11b is a macrophage surface protein, which is recognized as a macrophage marker. As shown in Figure 7F, the expression of CD11b in the serum increased dramatically in the blood of HFD-Ovx-treated ApoE^−/−^ mice. While E_2_ and catalpol significantly inhibited the expression of CD11b. The expressions of M1 macrophage markers (IL-12β, CD86, and iNOS) in aortic lysates were promoted by a high-fat diet, and they were increased further in HFD-Ovx-treated ApoE^−/−^ mice. The treatment with E_2_ and catalpol could effectively reduce the expressions of M1 macrophage markers (Figure 8A). Conversely, the decreases of M2 macrophage markers (FIZZ1, CD206, and Arg-1) in aortic lysates were caused by a high-fat diet and ovariectomy. Both E_2_ and catalpol reversed the declines of M2 macrophage markers (Figure 8B). Besides, the expression of M1 and M2 macrophage markers in the aorta cross-sections were detected by immunohistochemistry (Supplementary Figure S3). Then the peritoneal macrophages of mice were extracted and identified (Supplementary Figure 4). Similarly, catalpol down-regulated the expressions of M1 macrophage markers and up-regulated M2 macrophage markers in peritoneal macrophages (Figures 8C,D).

### Catalpol Promoted the Expressions of ERs in HFD-Ovariectomy-Treated ApoE^−/−^ Mice

**FIGURE 9:**
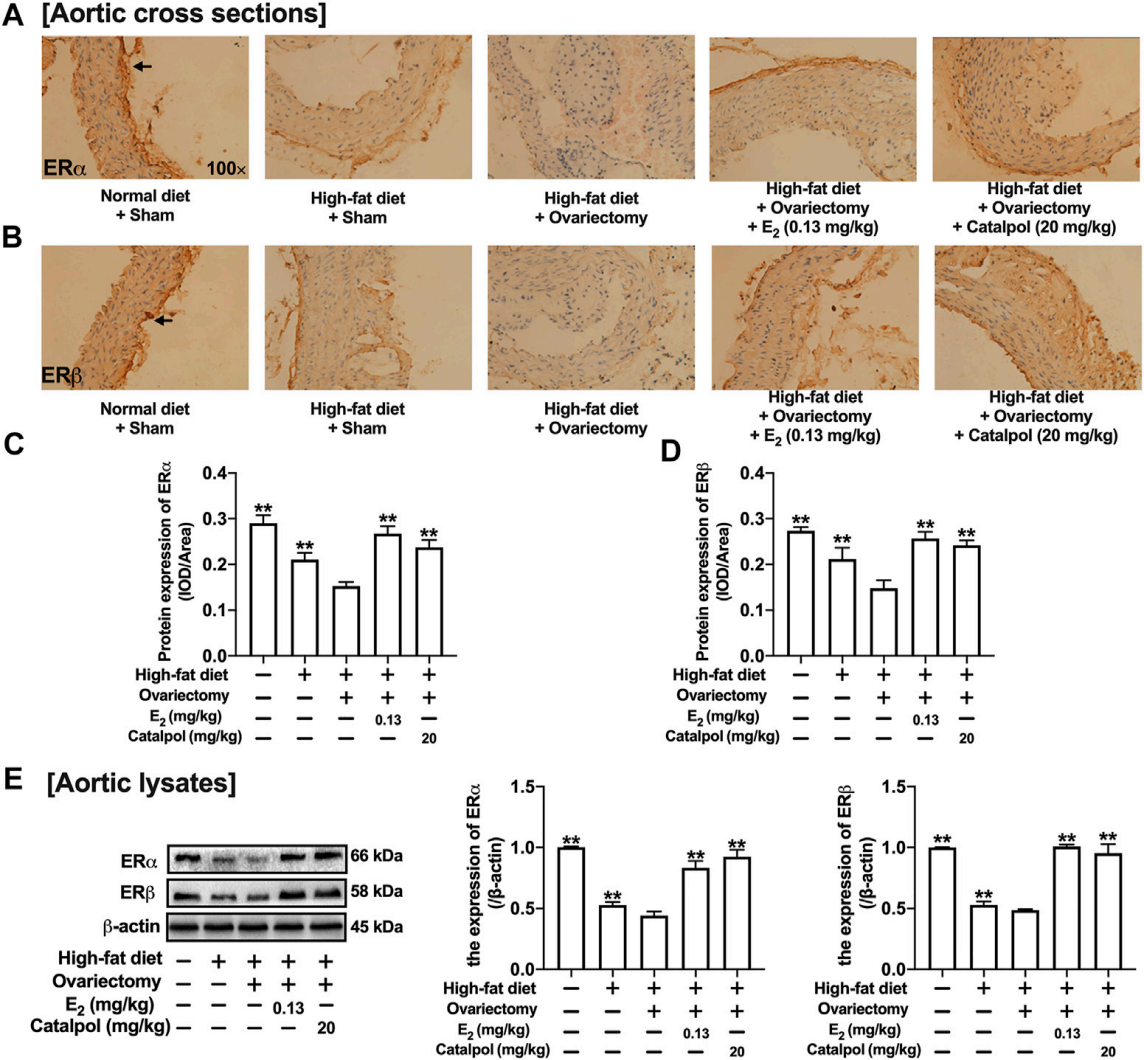
Catalpol promoted the expressions of ERs in HFD-Ovx-treated ApoE^−/-^ mice. Effects of catalpol on the expressions of ERα **(A)** and ERβ **(B)** in cross-sections of the aortas. **(C)** Effects of catalpol on the expressions of ERα and ERβ were also detected by western blot (*n* = 3). Data are expressed as mean ± SD. *p < 0.05, **p < 0.01 vs. Ovx group.

The ERs expressions of aortas were assessed by immunohistochemistry and western blot. As shown in Figures 9A–C, the expression of ERα in the aortic cross-sections was obviously inhibited by a high-fat diet, and it was reduced further in HFD-Ovx-treated ApoE^−/−^ mice. While the treatment with E_2_ and catalpol significantly promoted the ERα expression. Besides, the high-fat diet and ovariectomy led to the decline of ERβ expression in the aortic cross-sections, and the decrease was reversed considerably by E_2_ and catalpol (Figures 9B,D). As shown in Figure 9E, the results of western blot were consistent with immunohistochemistry. However, catalpol did not reduce the expression of ERβ in M1 macrophages. It suggested that other mechanisms were involved in the effect of catalpol on reducing ERβ expression in HFD-Ovx-treated ApoE^−/−^ mice.

## Discussion

The macrophage is a kind of immune cell that owns the multi-functional biological function. It can not only participate in the non-specific immune response by secreting inflammatory factors and phagocytic lipids, but also in the specific immune response through antigen presentation (Deligne and Midwood, 2021). The primary function of macrophages is to eliminate some cell fragments, lipids, or pathogenic microorganisms through phagocytosis *in vivo* (Zhou et al., 2021). At the same time, they can also secrete some cytokines to regulate immunity and maintain tissue homeostasis. Notably, there are some differences in biological functions between the two phenotypes of macrophages.

M1 macrophages are mainly distributed in the plaque area, which is prone to rupture. They can degrade the extracellular matrix by secreting several matrix metalloproteinases (MMPs), which cause instability and rupture in plaques (Krams et al., 2006). M1 macrophages have weak lipid transport and clearance ability during the AS process, while they own intense aggression and high antigen presentation ability. According to the microenvironment, M1 macrophages can activate other immune cells in the tissue, secrete pro-inflammatory factors, and accelerate AS (Chen et al., 2015). M2 macrophages are far away from the lipid core of plaque, and primarily distributed in the stable area of the plaque. M2 macrophages possess a solid ability to remove lipids by phagocytosis (Ng et al., 2021). They can secrete anti-inflammatory cytokines (such as IL-10, TGF-β) to help wound healing, which plays a significant role in tissue repair. In the early stage of AS, the plaques are mainly infiltrated by M2 macrophages. While with further aggravation, M2 macrophages have obstacles in the process of lipid clearance and gradually transform into the M1 phenotype (Colin et al., 2014). *In vivo*, our results revealed that the inflammation and damage of aortic vessels were aggravated in postmenopausal AS mice. The expressions of M1 macrophage markers were abundant in the aortic. Various environmental stimuli can cause the polarization of the M1 macrophage. The mixture of LPS and IFN-γ has been widely used to induce the M1 polarization model (Wang X. et al., 2019). According to the experiments, catalpol could reduce the polarization of M1 macrophage, suppress inflammation, stabilize the necrotic core, and avoid plaque rupture. However, the report claimed that CD86 was insufficient evidence to represent M1 macrophage Wang L.x. et al. (2019), more M1 and M2 markers should be involved in our future study (Jablonski et al., 2015).

There is an interaction between inflammation and oxidative stress. Inflammatory cells can produce ROS, which is involved in oxidative stress. On the contrary, ROS can activate NF-κB and increase of inflammatory cytokines (Bae et al., 2017; Kumar et al., 2018). Reducing the level of ROS suppressed the plaque formation in ApoE^−/-^ mice (Nomura et al., 2014). NO is an active substance produced and released by vascular endothelial cells. NO can dilate blood vessels, inhibit the proliferation of vascular smooth muscle (Lewis and Lang, 2015; Cheng et al., 2016). It can improve the function of vascular endothelial cells, thus inhibit the development of AS. At the same time, NO is regulated by two proteins (Forstermann and Sessa, 2012). The NO synthesized by eNOS can inhibit cell apoptosis and is considered a survival factor of endothelial cells. It has various biochemical activities, including clearing superoxide radicals directly, reducing leukocyte adhesion and activation, and maintaining endothelial integrity (Ueda et al., 2015). With the stimulation by inflammation and cytokines, iNOS can generate many NO, which can combine with oxygen and peroxides (Kapralov et al., 2020). It is a potent oxidative free radical. When the expression of iNOS increases, NO acts as a gas-free radical and aggravates cell injury and apoptosis (Zhu et al., 2016). Estrogen was reported to be able to produce eNOS through ERα and induce endothelium-dependent vasodilation. Also, the effect of estrogen was mainly mediated by PI3K/Akt cascade and MAPK (Hernandez-Resendiz et al., 2015). Our experimental results showed that the lack of estrogen could reduce the level of NO in the blood. However, the treatment with catalpol could increase the expression of eNOS and reduce the expression of iNOS.

**FIGURE 10:**
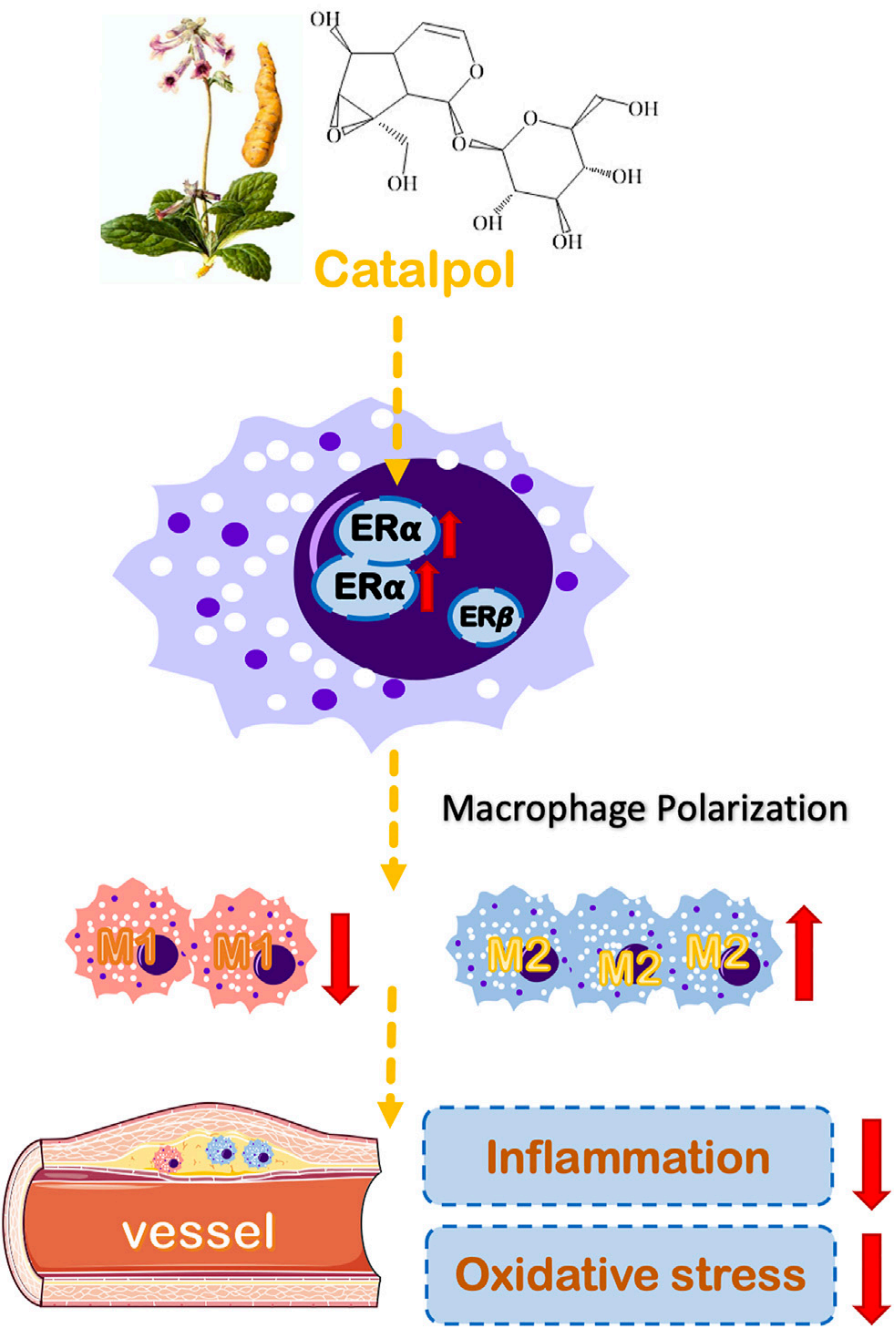
A schematic diagram describing the protective mechanisms of catalpol against macrophages polarization and postmenopausal AS through ERα.

Epidemiological studies have found that estrogen has a strong cardiovascular protective effect and can treat menopausal atherosclerosis by acting on endothelial cells and macrophages (Toniolo, 2014). The biological effect of estrogen is mainly mediated by ERα and ERβ (Paterni et al., 2014). ERα is the primary estrogen receptor expressed in mice macrophages (Campesi et al., 2017). The release of TNF-α induced by LPS was increased in macrophages of ERα knockout mice. Besides, the absence of ERα can promote macrophage dysfunction, which implies that ERα participates in the inflammatory response (Brown et al., 2010). In macrophages, estrogen can up-regulate the expression of ERα, but it has no significant effect on ERβ. Lacking ERα expression can block the protective effect of estrogen on immune response and AS. Our results showed that the expression of ERα was dramatically decreased in postmenopausal AS mice and M1 macrophages. Besides, the results of molecular docking implied that catalpol could bind with ERα (Supplementary Figure 5). The treatment with catalpol could significantly increase the expression of ERα rather than ERβ. Then, both ERα and ERβ inhibitors were applied. With the ERα inhibitor, the anti-oxidation and anti-inflammatory ability of catalpol disappeared. It confirmed that catalpol prevented postmenopausal AS and macrophage polarization through activating ERα, rather than ERβ (Figure 10).

Clinical studies have found that estrogen replacement therapy has many adverse reactions, which increases the risk of breast cancer in women (Boukhris et al., 2014). Therefore, it has become an urgent need to find some more safe estrogen replacement therapy. Many natural plants have phytoestrogens, similar to estrogen in structure and function (Al-Anazi et al., 2011). They can up-regulate the expression of estrogen receptors and have an estrogen-like effect. Generally, our present study gave evidence that catalpol could regulate macrophage polarization by increasing the expression of ERα. It may provide new insight into the molecular mechanisms for catalpol in preventing postmenopausal AS. However, with the aim of developing catalpol as an eligible therapeutic agent in the clinic, more mechanisms of catalpol involved in preventing postmenopausal AS are required to be detected in future studies.

## Data Availability

The raw data supporting the conclusions of this article will be made available by the authors, without undue reservation, to any qualified researcher.

## Abbreviations

AS, atherosclerosis; CRP, C-reactive protein; DMED, Dulbecco's modified Eagle's medium; E2, 17β-estradiol; eNOS, endothelial nitric oxide synthase; ERs, estrogen receptors; FBS, fetal bovine serum; HDL-C, high-density lipoprotein cholesterol; H&E, hematoxylin-eosin; IFN-γ, interferon-γ; IL, interleukin; iNOS, inducible nitric oxide synthase; LDL-C, low-density lipoprotein cholesterol; LPS, lipopolysaccharides; MMPs, matrix metalloproteinases; MPP, methyl-piperidino-pyrazole; Ovx, ovariectomy; PCR, polymerase chain reaction; PHTPP, 4-(2-phenyl-5,7-bis[trifluoromethyl]pyrazolo[1,5-a]pyrimidin-3-yl)phenol; ROS, reactive oxygen species; TC, total cholesterol; TG, triglyceride; TGF-β, transforming growth factor-β; TLR4, toll-like receptor 4; TNF-α, tumor necrosis factor α.
