# Supplementary figures and images for: Catalpol Inhibits Macrophage Polarization and Prevents Postmenopausal Atherosclerosis Through Regulating Estrogen Receptor Alpha
Source: Front Pharmacol. 2021 Apr 30;12:655081. doi: 10.3389/fphar.2021.655081 (PMC8120111; doi:10.3389/fphar.2021.655081)

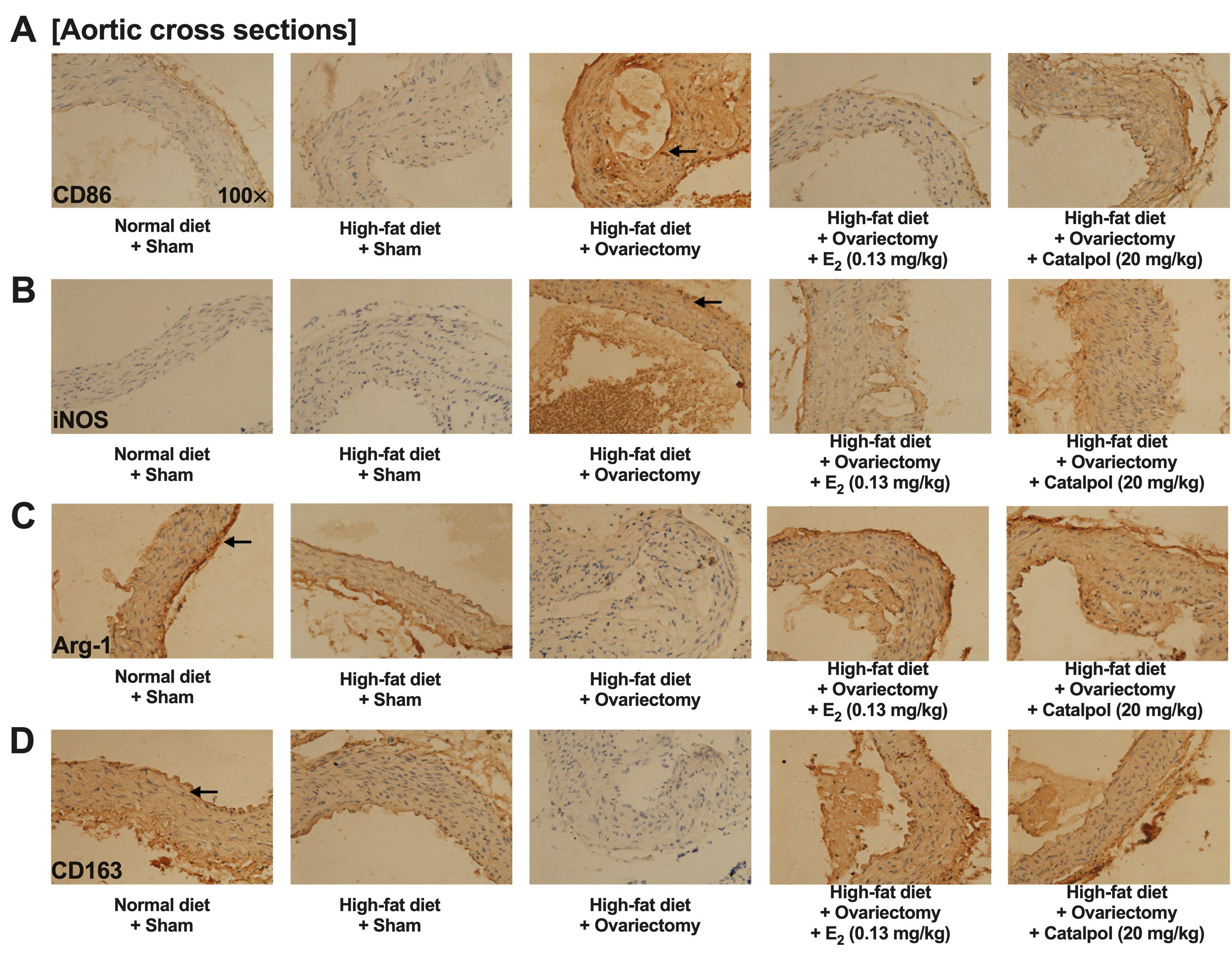

Supplement: Supplementary file 1 [file Image3.TIFF]

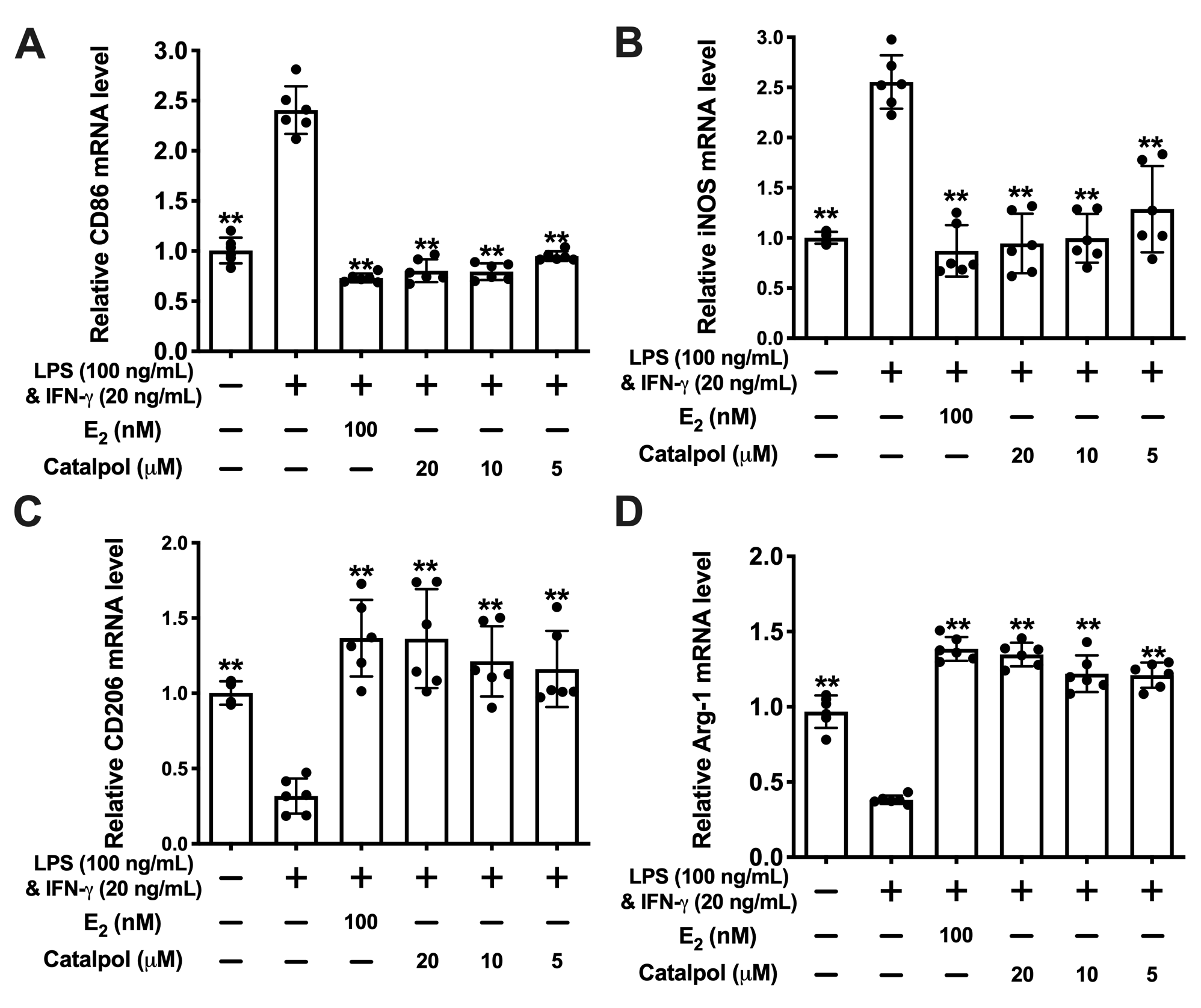

Supplement: Supplementary file 2 [file Image1.TIFF]

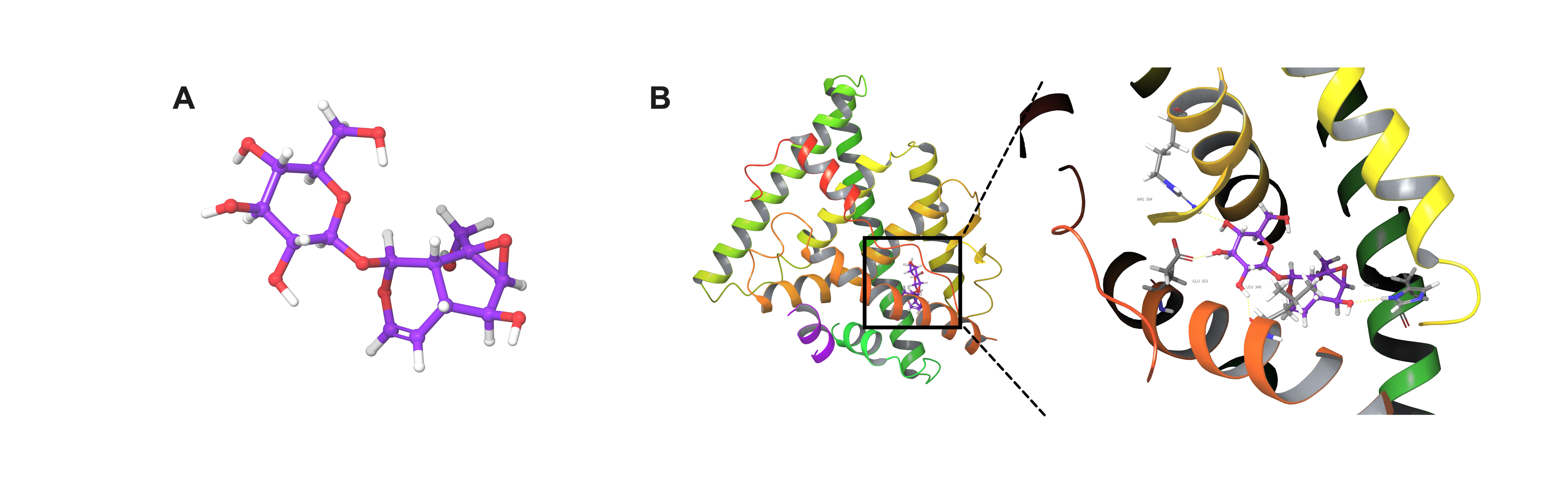

Supplement: Supplementary file 4 [file Image5.TIFF]

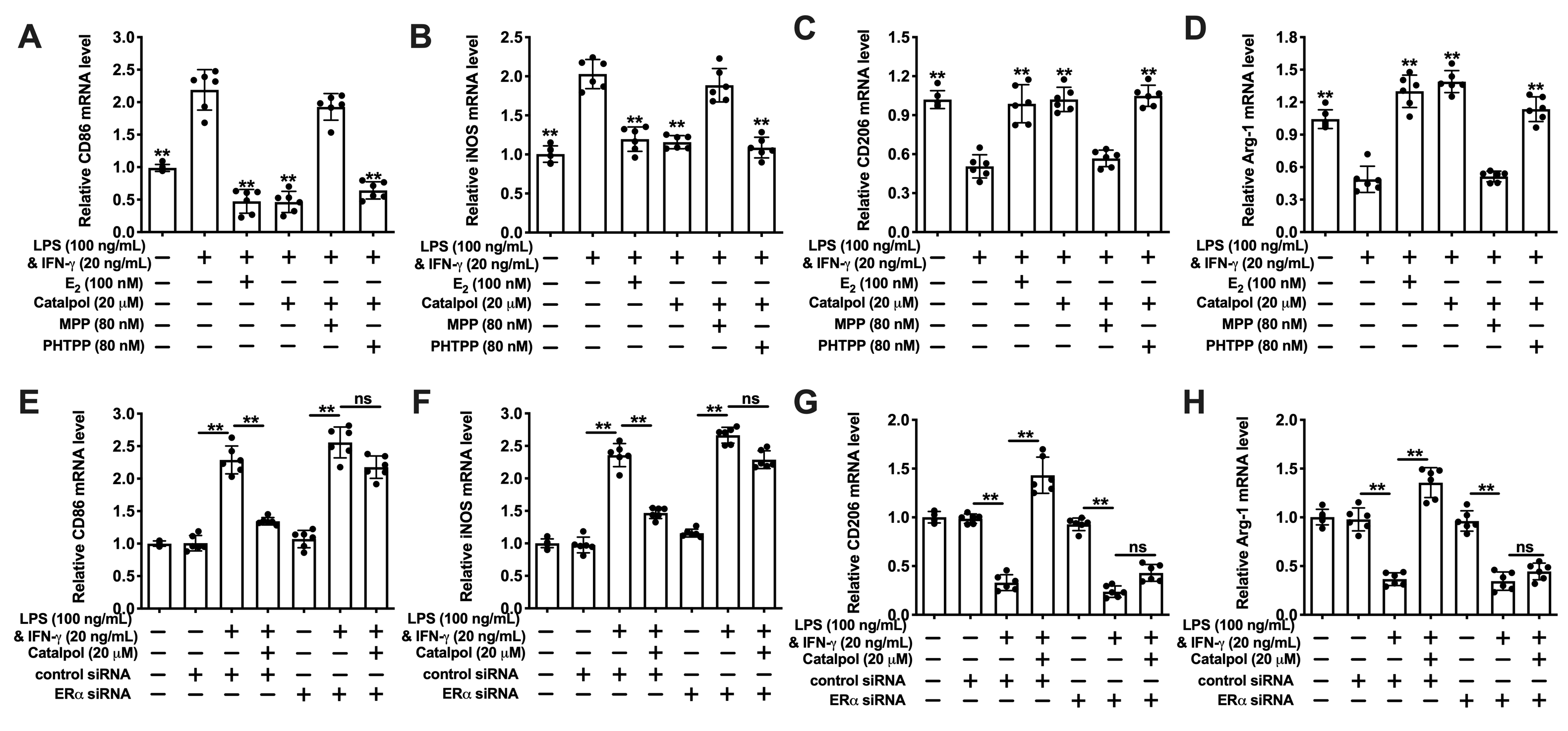

Supplement: Supplementary file 5 [file Image2.TIFF]

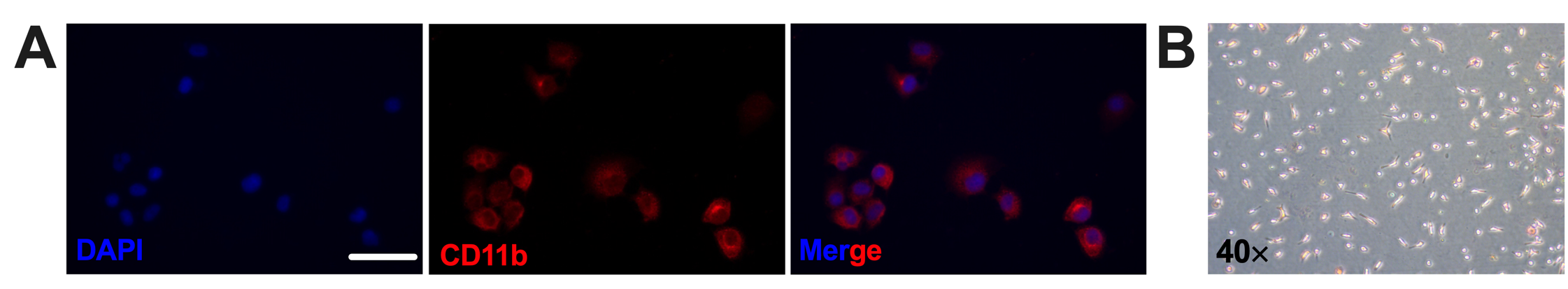

Supplement: Supplementary file 6 [file Image4.TIFF]
