# Supplementary material for: Catalpol Inhibits Macrophage Polarization and Prevents Postmenopausal Atherosclerosis Through Regulating Estrogen Receptor Alpha
Source: Front Pharmacol. 2021 Apr 30;12:655081. doi: 10.3389/fphar.2021.655081 (PMC8120111; doi:10.3389/fphar.2021.655081)
Supplement: Supplementary file 3 [file Table1.DOCX]

Table 1. The sequence of primers for real-time PCR

| **Gene Name** | **Primer Sequence（5' - 3'）** |
| --- | --- |
| CD206 | F: CTCTGTTCAGCTATTGGACGC |
|  | R: CGGAATTTCTGGGATTCAGCTTC |
| CD86 | F: TCAATGGGACTGCATATCTGCC |
|  | R: GCCAAAATACTACCAGCTCACT |
| Arg-1 | F: TGTCCCTAATGACAGCTCCTT |
|  | R: GCATCCACCCAAATGACACAT |
| iNOS | F: GGAGTGACGGCAAACATGACT |
|  | R: TCGATGCACAACTGGGTGAAC |
| GAPDH | F: TGGCCTTCCGTGTTCCTAC |
|  | R: GAGTTGCTGTTGAAGTCGCA |
